# Interpreting Degree Semantics

**DOI:** 10.3389/fpsyg.2019.02972

**Published:** 2020-01-30

**Authors:** Alexis Wellwood

**Affiliations:** School of Philosophy, University of Southern California, Los Angeles, CA, United States

**Keywords:** scale structure, truth conditional meaning, semantic anomaly, language and mind, compositional semantics

## Abstract

Contemporary research in compositional, truth-conditional semantics often takes judgments of the relative unacceptability of certain phrasal combinations as evidence for lexical semantics. For example, observing that *completely full* sounds perfectly natural whereas *completely tall* does not has been used to motivate a distinction whereby the lexical entry for *full* but not for *tall* specifies a scalar endpoint. So far, such inferences seem unobjectionable. In general, however, applying this methodology can lead to dubious conclusions. For example, observing that *slightly bent* is natural but *slightly cheap* is not (that is, not without a “too cheap” interpretation) leads researchers to suggest that the interpretation of *bent* involves a scalar minimum but *cheap* does not, contra intuition—after all, one would think that what is minimally cheap is (just) free. Such claims, found in sufficient abundance, raise the question of how we can support semantic theories that posit properties of entities that those entities appear to lack. This paper argues, using theories of adjectival scale structure as a test case, that the (un)acceptability data recruited in semantic explanations reveals properties of a two-stage system of semantic interpretation that can support divergences between our semantic and metaphysical intuitions.

## 1. Introduction

This paper examines a corner of semantic theory that has received a lot of attention in the recent linguistic and philosophical literature: the recruitment of ‘scale structure' in compositional accounts of the interpretation of sentences like (1) and (2)^1^.


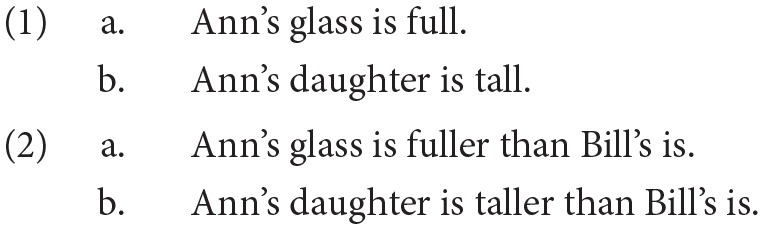


According to degree-based theorists, we can learn something about the meaning of (2) by thinking about how its parts compositionally determine truth conditions as follows: (2a) is true only if the degree to which Ann's glass is full is greater than the degree to which Bill's glass is full, and (2b) is true only if the degree to which Ann's daughter is tall is greater than the degree to which Bill's daughter is tall. Correspondingly, without specification of an explicit standard for comparison by a phrase like *than Bill's is*, (1a) is true only if the degree to which Ann's glass is full exceeds the contextually given standard for fullness, and (1b) only if where her daughter's height exceeds the relevant contextual standard; and so on.

Degree-semantic theories provide the rudiments for expanding outward the kinds of structures that can be compositionally interpreted with few additional assumptions, and they work to capture the kinds of inferences that we intuitively find to hold between relevant sentences. For instance, the simple appeal to a greater-than relation between degrees correctly predicts that if (2a) is true, then (3a) is false (evaluated in the same contexts) and, in turn, appealing to the intuitive idea that the negative of an antonymic pair reverses the ordering relation, correctly predicts that if (2b) is true so is (3b).


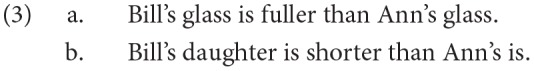


Very quickly, though, a theory that was primarily designed to offer perspicacious compositions that get the truth-value judgments right is leveraged to explain patterns of semantic anomaly. Here are some examples of the kinds of observations I have in mind. First, while it is possible^2^ to construct a comparative construction that targets two adjectives simultaneously, (4a), in many cases the result is anomalous, e.g., (4b)^3^.


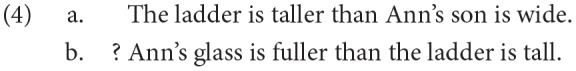


Second, while a modifier like *completely* can sometimes be used to indicate maximal extent along a given dimension, e.g., (5a), in many cases it cannot, e.g., (5b).


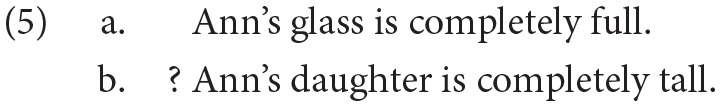


The going explanation for the asymmetry in (4) assumes that it is only possible to evaluate a comparative relation between two degrees if those degrees share a dimension; (4b), then, is anomalous because the scales associated with *tall* and *full* order degrees along different dimensions. The going explanation for (5) relates to the structure of the degrees so ordered: *completely* picks out the topmost, or maximal, degree on a scale; by hypothesis, the scale associated with *full* provides such an element, but that associated with *tall* does not^4^.

Let us take a closer look at these explanations. First, we observe that two strings of words that appear to be syntactically equivalent differ in acceptability. Next, we link these differences in acceptability to differences in the sorts of things that the expressions occurring in the sentence are about:^5^ *tall* is about length in the vertical dimension while *full* is about something else. Finally, depending on the target observation, different features of those things are recruited to explain the anomaly: *completely* relates to a scalar endpoint, and some dimensions (like vertical distance) apparently lack such points. Research can get off the ground and continue in stride without wondering much about what is meant when we say “what the expressions are about,” but, ultimately, we will want to know whether such explanations are correct or not. If it turned out, for example, that *tall* was not actually about vertical distance or that *full* actually was, or if it turned out that the scale of tallness in fact had an upper bound while the scale of fullness did not, that would certainly be problematic for the theory. But how can we tell what these scales are like, independently of the linguistic diagnostics?

The trouble facing such explanations is put into stark relief when we find clear examples where the linguistic tests turn up results that run counter to our intuitions about what there is (what we may call our *metaphysical intuitions*). For example, just as *completely* is thought to be licensed by gradable adjectives that are associated with scalar maxima, it is contended that *slightly* is licensed with gradable adjectives that associate with scalar minima. In this light, consider the asymmetry in (6).


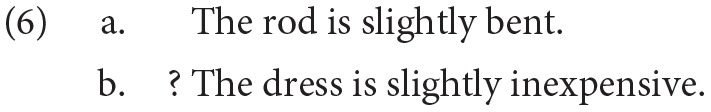


The explanation for (6) should run as follows: since *bent* is associated with a scalar minimum, *slightly* is licensed in (6a); but since *cheap* does not so associate, *slightly* is not licensed in (6b). But this seems odd: if we would otherwise suppose that the scale of inexpensiveness is, or is isomorphic to, a scale of cost, there should be a minimum element that is simply 0 dollars (or whatever). We have here a mismatch between the acceptability data and our intuitions about what there is; yet the explanation for the former would seem, on extant accounts, to depend on facts about the latter^6^.

Moreover, such cases can be multiplied. von Stechow (1984) and Rullmann (1995) suppose that (7) is deviant because *tall* associates with a scale that has no maximum. [This account dovetails, of course, with the expectations of other authors' interpretations of the fact that *completely* is anomalous with *tall*, recall (5b)].


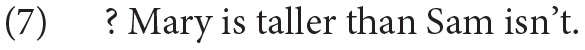


However, the scale associated with *full* apparently does have a scalar maximum [recall (5a)], yet (8) is deviant (cf. Lassiter 2011, p. 12).


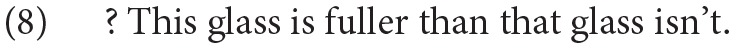


Lassiter furthermore points to adjectives like *tall* that are intuitively lower-bounded yet fail to pass tests for minimal scalar points. For example, compatibility with *slightly* is meant to track this scalar property, and yet, if that is the right analysis (see footnote 6), neither of the phrases in (9) mean what they should mean. That is, certainly neither (9a) nor (9b) should give rise to any felt anomaly, all else being equal; and, it seems to the current author, (9a) should just mean that Ann is really, really short, and (9b) that the watch is really, really cheap.


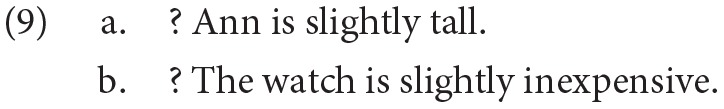


These issues did not go unnoticed by Kennedy (2007), who writes (p. 34–5),

…why do the scales used by particular adjectives have the structure they do? For example, naive intuition suggests that the cost scale should have a minimal value representing complete lack of cost, just as the dirt scale has a minimal value representing complete lack of dirt. However, the unacceptability of *??slightly/??partially expensive* and *??perfectly/??completely/??absolutely inexpensive* (cf. *slightly/partially dirty* and *perfectly/completely/absolutely clean*) indicates that as far as the gradable adjective pair *expensive/inexpensive* is concerned, this is not the case: the scale used by these adjectives to represent measures of cost does not have a minimal element.…The structure of a scale is presumably determined mainly by the nature of the property that it is used to measure, but the different behavior of e.g., *expensive/inexpensive* vs. *dirty/clean* suggests that this aspect of linguistic representation may diverge from what naive intuitions suggest.

Here, Kennedy raises the possibility of a divergence between intuitive judgments regarding the properties that are “out there” and their linguistic representations, whatever those might turn out to be.

Semanticists differ in their degree of comfort with this state of affairs. Lassiter (2010, p. 205) appears be worried, since: given our “intuitive assumptions about the nature of the scales in question, [the sentences in (10)] should be equivalent,” contrary to fact.


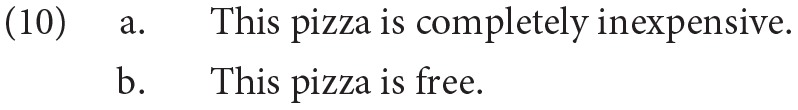


Klecha (2012), in contrast, is not worried: in his discussion of the scale structure associated with the epistemic adjective *likely*, he writes that “ultimately the ‘intuitive scale' associated with an adjective does not always align with its lexical scale” but “nor should we expect it to”; instead, we may acknowledge what “may seem like counterexamples,” but, “just because these intuitive scales” have some apparent bounding property, “we should not conclude that the lexical scales” do too (p. 11)^7^.

In general, the position expressed most stridently here by Klecha is common in linguistic semantics, but I have only found it discussed explicitly in the context of evaluating whether natural language semantics is best pursued as a theory that interfaces with metaphysics as opposed to something else. The predominant view arising in these discussions appears to be that instead of building a theory of how linguistic expressions compositionally relate to the (real) world, we build a theory of how linguistic expressions compositionally relate to the world as we talk about it. Therefore, we assume a world that is as language suggests it to be, not as it actually is, and the interfacing theory for semantics is “natural language metaphysics” rather than (real) metaphysics (Bach, 1986; Bach and Chao, 2012; cp. Moltmann, 2017). The problem with such a position, I contend, is that it amounts to a refusal to say what semantics properly interfaces with; under these conditions, its theoretical statements cannot be evaluated for truth and falsity. This renders semantics non-scientific.

More generally: if a semantic theory aims to explain certain semantic judgments in terms of something else—such as what those expressions are about—then it had better be that we have an independent theory of what expressions are or can be about. In other words, the theory has to respect both our semantic and metaphysical intuitions and provide for a way of resolving mismatches where they are found. Much caution is warranted. For present purposes, relevant modifiers and gradable adjectives might be polysemous^8^, and in ways that are not entirely predictable; this requires antecedent caution in interpreting the results of our linguistic tests. And, even supposing that we can fix on the appropriate senses for the purposes of making judgments, not all of the tests work all the time, “for apparently idiosyncratic reasons” (Kennedy, 2007, p. 34).

I think there is a way to account for semantic anomaly and to respect our independent judgments of what our expressions are about. However, much of the hard work of showing how to do it has not yet been done. This paper will not do all of that work, but it aims to contribute to the bigger project by focusing in on explanation in this corner of degree semantics. Section 2 gives a number of additional examples of theoretical posits proffered within that framework and describes some of the explanations those posits are used in service of. Section 3 returns to the question of what we understand our semantic theory to be doing—whether relating expressions to the (real) world, to the world as we talk about it, or, instead, to other areas of cognition. And, section 4 takes a stab at a specific positive proposal. This proposal—interpretation in two steps—holds some additional appeal in that it provides some resources for capturing polysemy.

## 2. Matters for Interpretation

This section briefly lays out the essentials of a degree-based compositional semantic theory, with special attention to the hypothetical nature and variety of things that linguistic expressions are about as they are recruited for semantic explanation^9^. These roughly fall into three categories that are not entirely independent: degrees, the scalar relations that order them, and the measure functions that relate entities to scales. I lay out some of the claims here but will not, for the most part, comment on their interrelations.

Beginning with degrees themselves, a first distinction found in the literature is between whether degrees should be understood to be primitive (i.e., not reducible to abstractions based on other objects; the default assumption) or as labels for equivalence classes of objects (as in Cresswell, 1976), possible objects (Schwarzschild, 2013), or of states (Anderson and Morzycki, 2015), etc. Appeal to degrees simpliciter, or to aspects of their nature, has important consequences for the data coverage of a degree-based theory. Additionally, while I will not discuss it here, their importance for linguistic theory is supported by the need for an account of movement-like properties in *than*-clauses, which receives a natural account in terms of abstraction over degrees; see Kennedy (2002) for extensive discussion and references.

With the introduction of degrees, we are able to explain certain basic data concerning the interpretation of comparatives with *-er*/*more, as*, etc. In a degree semantic setting, such comparative constructions are typically analyzed in terms of a greater-than relation between two degrees *d* and *d*′, such that *x* *is* *A-er than* *y* is true only if *x* is mapped to a higher degree on the scale associated with *A* than *y* is. Some adjectives associate with the same scale, or so it is supposed based on consideration of what have come to be called “subcomparatives” like (4), repeated as (11) below.


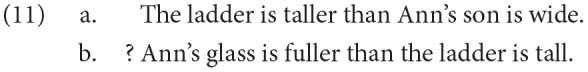


These examples show that while distinct adjectives *A* and *A*′ occur in the matrix and *than*-clauses of the comparative, not everything goes: comparatives like *x* *is* *A-er than* *y* *is (A*′*)* are true just in the case where *x* is mapped to a higher degree on the scale that is common to *A* and *A*′ than *y* is^10^. Since (11a) is perfectly acceptable and interpretable while (11b) is not, we may posit thereby that (11a) involves adjectives that share a scale of length whereas there is no common scale for (11b).

A second cut is in whether the comparative morphology relates degrees simpliciter (i.e., degrees as points, ordered by some ≤) or convex sets of such degrees (i.e., degree intervals, ordered by an inclusion relation ⊑). Interpreting comparatives as essentially relating scalar intervals helps to explain otherwise puzzling data relating to quantificational noun phrases in *than*-clauses (see especially Schwarzschild and Wilkinson, 2002; Fleisher, 2016). Assume that we have Ann and 10 other people, such that 5 people are shorter than Ann and 5 people are taller. Under these circumstances, (12) is intuitively false.


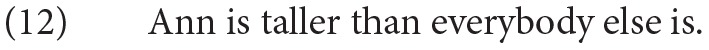


Yet, supposing that the derivation of *the* degree named by a *than*-clause involves some calculation using a set like {*d*: everybody but Ann has *d*-height}, there is no way to predict this judgment correctly; this difficulty can be overcome by positing that the calculation involves certain sets of degrees (see Schwarzschild and Wilkinson, 2002 for details).

Kennedy (2001) builds on the idea that comparative constructions involve the manipulation of scalar intervals but extends it so that these may come in positive and negative varieties^11^. In particular, he aims to account for the fact that, even if two adjectives share a dimension, the comparative form is unnatural if the two adjectives are opposite in polarity; see, for instance (13).


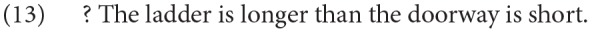


Kennedy explains the anomaly of examples like (13) by positing that *long* relates the ladder to a positive interval—one stretching from 0 length to the length of the ladder—while *short* relates the doorway to a negative interval—one stretching from the length of the doorway up to infinity. Since there is, in principle, no possible inclusion relation between such degrees, Kennedy suggests, the comparative form is anomalous^12^^,^^13^.

More can and has been said about degrees per se, but present purposes concern what has been said of the scales that order them. The most lauded aspect of scalar structure in the degree semantics literature in recent years concerns whether the relevant scale has certain privileged elements—an upper bound or maximum and a lower bound or minimum (Rotstein and Winter, 2004; Kennedy and McNally, 2005). A battery of tests, some of which were cited in the previous section, are thought to diagnose whether an adjective or antonymic pair associate with different scales in a typology like that displayed in (14), where the examples given are instances of hypothesized antonymic pairs whose shared scale bears the relevant properties (from Kennedy and McNally, 2005).


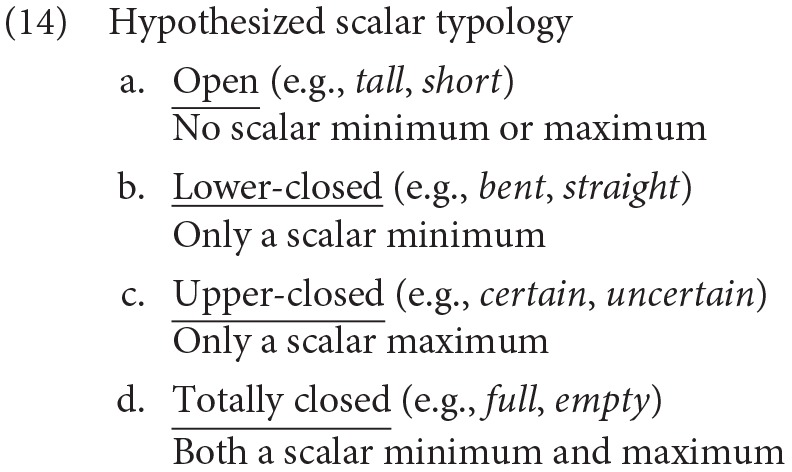


One last arena in which degree semantics makes substantial demands of ontology or conception in its explanations concerns measure functions, which introduce a relation between measured entities and the scales that represent their measures^14^. Given basic assumptions of degree-semantic theories, we need not expect any particular correspondences between (call it) the structure of the entities measured and that of the scales used to measure them. And while it may appear that we do not see such a correspondence, in some cases we certainly do.

For one such case, consider the comparatives in (15).


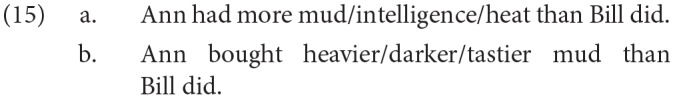


With bare *more* in (15a)^15^, the meaning of the noun determines dimensionality: *more mud* can be used to express a thought about relative volume or weight, but not about heaviness, darkness, or tastiness, unlike (15b). Meanwhile, *more intelligence* and *more heat* cannot, or so it seems, be used to express a thought about relative volume, weight, heaviness, darkness, or tastiness, etc; rather, their dimensions are specific to whatever *intelligence* and *heat* describe.

The facts are parallel in the verbal domain; consider (16).


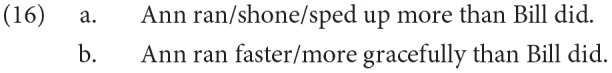


To say that (15a) involves instances where there must be alignment between what is measured and how it is measured (i.e., what scale is used to represent the measurement) is to say that the dimensions for comparison with bare *more* uniformly appear to preserve certain formal features that the measured domains appear to have. That is, many authors have described the relevance of mereological or part-whole relations on the extensions of (at least) phrases like *mud* and *run*: whatever *mud* can be truthfully used to describe, it also can truthfully describe arbitrary subparts thereof (Cartwright, 1975; Link, 1983, and many others); and whatever *run* truthfully applies to, it also truthfully applies to arbitrary subparts thereof (Taylor, 1977; Bach, 1986, and many others). These patterns of application can be modeled by partial orders on portions of mud or stretches of running, and it is the strict ordering relations that are preserved in the mapping to degrees (Schwarzschild, 2002, 2006; Nakanishi, 2007): smaller portions of the mud have smaller volume or weight measures but not smaller temperature measures; smaller stretches of running activity measure less by duration or distance but not by speed.

This is not the only arena in which structure-preserving relations between distinct ontological or conceptual domains have been important in degree semantics (see Hay et al., 1999; Kennedy and McNally, 2005; Kennedy and Levin, 2008; Piñón, 2008). It has been supposed that there are non-trivial correspondences between the scale structure associated with a gradable adjective and the telicity profile of its corresponding deadjectival verb. Of particular interest for present purposes is the observation that telic verbal descriptions track scalar maxima associated with their adjectival core (if available) while atelic verbal descriptions track derived scalar minima (see Kennedy and Levin, 2008 for discussion and references).

Relevant data include ‘degree achievements' (Dowty, 1979). Among the pertinent observations are: (i) some deadjectival verbs show variable telicity, and (ii) some are only atelic. With respect to (i), verbs such as *to cool* are said to be variably telic in that they are compatible both with telic (*in X time*) and atelic (*for X time*) modifiers. Interestingly for our purposes, depending on the modifier they show different implications: (17a) with a telic modifier suggests that the soup became maximally cool, while (17b) with an atelic modifier merely implies that the soup became cooler than it was before.


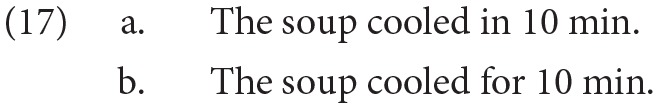


With respect to (ii), degree achievement verbs like *to widen* are only acceptable and interpretable with atelic modifiers, requiring only a minimal change in degree; compare (18a) and (18b).


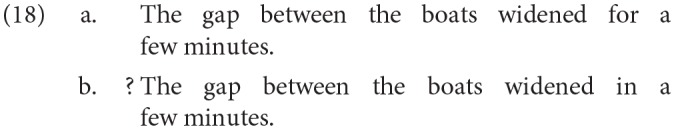


In Kennedy and Levin's analysis (see also Kennedy, 2012; McNally, 2017), the truth of such predications depends on the positive interpretation of their adjectival core; their truth conditions, in turn, are derived via a mapping from the scalar structure associated with the adjective into the event structure associated with its embedding verb phrase. In particular, variably telic predicates involve interpretation relative to scalar maxima (telic) or contextual standards (atelic). As in the positive adjectival form, whether the predication is maximal or not depends, by default, on whether the adjective's scale has a maximal element. Crucial for our purposes is the idea that the scale associated with the base adjective, call it *S*_*A*_, is mapped onto a scale, call it *S*_Δ_*A*__ that measures degree of change. These derived scales, it is supposed, all have a minimal element (corresponding to the degree of the object along *S*_*A*_, before the change occurs), but they only have a maximal element if *S*_*A*_ has a maximal element.

Thus, *to cool*, based on the upper-closed *S*_*cool*_ (witness the acceptability of *completely cool*), can be interpreted as telic—where an object *x* reaches the maximal degree of change possible along the relevant dimension, namely when *x* has reached the maximal degree of coolness—or atelic—where *x* reaches some change greater than the minimum, that is, where *x* was along *S*_*A*_ at the initiation of the change event. In contrast, *to widen* has only the atelic interpretation because the scale measuring change has exactly that kind of minimum—the degree to which *x* is wide at the start of the change event—but it fails to inherit a maximum from where it fails to exist in *S*_*A*_.

What should be clear is that quite a lot of the theoretical description of what is going on in this corner of language involves assumptions about the sorts of things quantified (degrees), how they are ordered, and the presence or absence of “special elements” in those orderings (scales), in addition to structure-preserving relationships between the degrees used to represent measurement and the entities so measured. What I want to know is: apart from the evidence of semantic analysis itself, however copious that evidence, what independent tests are there for the adequacy of the attendant semantic explanations? Precisely to the extent that those explanations depend on independent features of ontology or conception, we require the details from an independent theory that describes those features.

## 3. The Meaning Relation

For concreteness, let us regard some of the statements formalizing the theoretical claims presented in the previous section. For instance, Kennedy and McNally (2005) derive the interpretation of an adjectival phrase consisting of a gradable adjective like *expensive* and the silent positive morpheme, pos (responsible for linking entities with a contextual standard for the target adjective; see discussion and references in their paper). In addition to its role in selecting a standard in *c* for the adjective, itself interpreted as in (19a), pos has the function of binding the degree argument introduced by that expression, (19b).


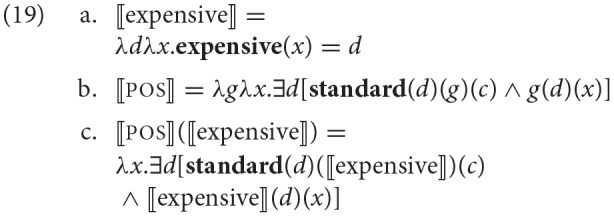


The result of this local computation is the property in (19c): it is a property true of individuals who measure some degree of expensiveness greater than the standard for expensiveness in *c*. The general schema in (20) highlights where and how the scale structure associated with the adjective might come into play: as the degree relation (e.g., the interpretation of the gradable adjective) acts as an argument to the **standard** function, which, for reasons described in Kennedy and McNally's paper, will default to the maximum when the degree relation has a maximum degree in its range, etc.


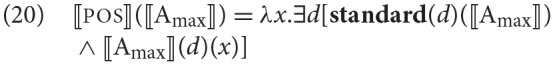


Kennedy and McNally, like the other authors whose work is considered in any detail here, assume a semantic framework like that laid out in Heim and Kratzer (1998), which is properly read as implying nothing more than a computational-level description of what speakers know when they can be said to know some piece of their language. Statements like those in (19) and (20) reflect a hypothesis about what such speakers know: they know the correspondences established by the interpretation function, ⟦·⟧. The manner of specification for the terms “on the right” of equations involving ⟦·⟧ usually are not intended to be taken as theoretically loaded qua symbols (see Dowty, 1979; Williams, 2015 for discussion of “semantic representations”). Nonetheless, if knowing one's language implies knowing such statements, and if such statements involve properties of things which are not obviously properly linguistic, then the theory depends for its explanations on the independent determination that those things in fact have those properties and that competent speakers know this^16^.

How do we determine whether the relata “on the right” have the properties our theories need them to have? There are different ways one can approach this question and thus different ways one may begin to get beyond the explanatory impasse. The first bites the bullet and supposes that semantic theory interfaces with research in metaphysics—the study of what there is. The second is impervious to the bullet and supposes that semantics proceeds in isolation, describing and depending on properties of things needed for semantic analysis but without any attendant commitment to whether those things have any independent existence, whether “out there” (metaphysical) or “in the head” (cognitive). The third dodges the bullet by supposing that, despite our theoretical talk of establishing word-world relations, our theory is primarily geared toward describing a relation between expressions and elements of non-linguistic cognition.

I will suggest that pursuing the third option provides our best hope of overcoming some of the challenges posed by our case study.

### 3.1. Language and the World

Taking the semantic theory to be truth-conditional—i.e., as specifying, for each well-formed sentence *S* of the language, what it would take for *S* to be true, in this or some possible world—takes it to depend, in non-trivial ways, on what is or could be true (see Travis, 1996). What does this mean for present purposes? Given a sentence *S*—say, *Ann's glass is completely full*—the theory pairs *S*, via ⟦·⟧, with a statement to the effect that *S* is true only if the scale associated with *full* has a maximal point, *d*_max_, and Ann's glass measures full to *d*_max_^17^. Among other things, this theory entails that there exists a scale of fullness that has certain properties. To some ears, this may sound straightforward and unimpeachable. However, if counter-examples like those discussed in section 1—those showing mismatches between our intuitions about which sentences are anomalous and what scales are like—are thoroughgoing and pervasive enough, a theory that depends on “what there is” for its evaluation may quickly come under threat.

The idea that “the meaning relation” establishes how expressions are related to the things we use our expressions to talk about has unobjectionable roots. First, as speakers, we use language to talk about the world, and the primary source of evidence for semantics comes from the correspondences (or lack thereof) between the way the world is and how we use our sentences to say that it is. Second, as theorists, we follow Lewis's and Cresswell's advice (by way of Partee, 1995): we broker the mystery of meaning by finding something that does what meanings do and study that; and, minimally, meanings make a difference in truth; so, we should be able to inform any study of meaning by way of the study of truth conditions.

A general problem is that specifying “the conditions under which *S* would be true” will involve specifying far more than what we want to attribute to the linguistic object, *S*, alone—and that is what a semantic theory aims to target. The trouble is easy enough to see in puzzle cases: considering the anomaly of a sentence like *The rock thinks it's raining*, Chierchia and McConnell-Ginet write, “the oddness seems linked more to the structure of the world than to facts about linguistic meaning: rocks just aren't the kind of thing that thinks, as it happens, but this seems less a matter of what *rock* and *think* mean than a matter of what rocks and thinking are like” (p. 48). But it is also plain in mundane cases: detailing the conditions under which *It was raining outside at noon on 10/4/2019* would be true would require, in fact, a catalog of how the whole world at a particular moment (and the history leading up to that moment) came to instantiate the state of affairs said to have been instantiated.

Returning to the central problem: how can we know what the properties of the things “on the right-hand side” are like such that we can evaluate our semantic theory for its own truth or falsity? Considering the theory to relate expressions to the world, we have two options for independent theories that might do the job of independently specifying worldly properties: physics or metaphysics. If a criterion for a semantics-as-science is that its interfacing theory is empirical, then we should go with physics. But this will not do; as Hobbs (1985) succinctly puts it (p. 20),

Semantics is the attempted specification of the relation between language and the world. However, this requires a theory of the world. There is a spectrum of choices one can make in this regard. At one end of the spectrum—let's say the right end—one can adopt the “correct” theory of the world, the theory given by quantum mechanics and the other scientists. If one does this, semantics becomes impossible because it is no less than all of science, a fact that has led Fodor (1980) to express some despair. There's too much of a mismatch between the way we view the world and the way the world really is. At the left end, one can assume a theory of the world that is isomorphic to the way we talk about it. …In this case, semantics becomes very nearly trivial.

I do not think semantics is trivial. But how do we ensure that it is not? When semanticists are explicit about the question of an independent, interfacing theory, they tend to assert that we do not need one and that we can do just fine with a model of things we “talk *as if* ” there are. But this will not do either, as I discuss next. A quite different alternative, of course, would involve reinterpreting the statements in our semantic theory as reflections of (or abstractions over) how our expressions relate to categories of mind; I discuss this in section 3.3.

### 3.2. “Talk *as if*”

Some contend that the entities posited in semantic explanations have an existence entirely within the theory and do not (and should not) depend for their properties on an independent theory^18^. Thus, semantics traffics in what we talk *as if* there is (Bach, 1986; Bach and Chao, 2012; cp. Moltmann, 2017) and understands that talk neither in metaphysical nor cognitive terms. This position has come to be called “natural language metaphysics” (NLM; Pelletier, 2011 calls it “semanticism”). This position does have points in its favor, as reviewed below. But none of these overcome its inherent scientific deficiency.

For Pelletier (2011), the main considerations in favor of “talk *as if* ”/NLM have to do with apparently extensionally equivalent referents for terms that otherwise have been thought to be loaded with ontological commitment. For example, many truth-conditional approaches to the mass/count distinction suppose that it is characteristic of mass terms like *water* that they refer divisively, while count nouns like *cup* lack this sort of reference; as a reminder, for anything that *mud* applies to, *mud* also applies to any of its arbitrary subparts (mass; divisive reference), but for anything that *a toy* applies to, *a toy* does not also apply to its arbitrary subparts (count; non-divisive reference). In mereological approaches to the mass/count distinction (e.g., Cartwright, 1975; Parsons, 1979; Link, 1983), these referential profiles are attributed to ontological differences between what we might intuitively think of as “substances” and “objects.”

Pelletier (2011) takes issue with this because it just does not seem that a semantics based on wholes and subparts ad infinitum for the mass noun *water* jives with what we know about the stuff, water. After all, water has smallest parts—H_2_O molecules. He writes (p. 26)^19^.

A standard defense of the divisiveness condition in the face of these facts is to distinguish between “empirical facts” and “facts of language.” It is an empirical fact that water has smallest parts, it is said, but English does not recognize this in its semantics: the word *water* presupposes infinite divisibility.

It is not clear that this is true, but if it is, the viewpoint suggests interesting questions about the notion of semantics. If *water* is divisive but water isn't, then water can't be the semantic value of *water* (can it?). In turn this suggests a notion of semantics that is divorced from “the world”, and so semantics would not be a theory of the relation between language and the world. But it also would seem not to be a relation between language and what a speaker's mental understanding is, since pretty much everyone nowadays believes that water has smallest parts. Thus, the mental construct that in some way corresponds to the word *water* can't be the meaning of *water* either.

I will address the specific concern about there being a unique construct that *water* associates with in section 4. But Pelletier cites other empirical considerations that, he contends, militate semantic theory toward agnosticism: for one, in English and in other languages there are pairs of words that are drawn from the mass and count sides of the distinction and yet “the items in the world that they describe seem to have no obvious difference that would account for this” (p. 26), like *spaghetti* and *noodles*. And do we really think about what is on the plate differently depending on the word we choose? For another, citing data from Chierchia (1998), Pelletier notes that while both English and Italian have both mass and count noun forms corresponding to *hair/s*, in English you say *I cut my hair* but in Italian you say (the equivalent of) *I cut my hairs*; yet, clearly “It would seem that the same activity is described no matter where the barber is doing the work” (p. 29).

As an aside, I think there are reasons to suppose that these problems in particular do not loom as large as might seem, particularly if one posits a derivational—rather than merely lexical—account of the distinction between mass and count occurrences of nouns (cf. Borer, 2005). If *mud*, for example, amounts to meaning “stuff that we call mud” and *muds* amounts to “a plurality of entities, each of which is constituted by some stuff that we call mud,” do some of these worries evaporate? Regardless, it is unsatisfying in the extreme to conclude that we should thereby land firmly on the side of NLM, where nothing worldly nor conceptual should be recruited in order to help explain the mass/count distinction. After all, there certainly are robust correlations between the grammatical mass/count distinction and the notional object/substance distinction that will need explaining (cf. Rips and Hespos, 2015).

NLM amounts to a refusal to say what the interfacing theory with semantics is or should be. It thus puts semanticists in an uncomfortable place: assuming, as most do, that our compositional theories are bounded “on the left” by syntactic and morphological theory, we nonetheless resist bounding our theory “on the right” by anything at all. If there was nothing else that could be said, so be it. But it cannot be that we avoid committing simply in order to avoid making bad predictions.

### 3.3. Language and the Mind

What is left? What remains is the view that the study of language begins with its study as a faculty of mind and characterizes the knowledge recruited by that faculty during linguistic understanding and production. Semantic theory bridges the language faculty with other faculties of mind. On such a view, semantics tracks morphosyntactic structure “on the left” and non-linguistic cognition or conceptual structure on the right^20^. (Then, if we are lucky, the concepts and categories “on the right” can be, in their turn, related to aspects of real reality.)

This view requires, of course, that the theory take the form of a relation between two levels of structured representation. Computation in any form is syntactic, and the nature and structure of the symbols computed over play an important role in what computations can be performed^21^. When Lewis (1970) famously dismisses early attempts to characterize semantic theory as a relation between two languages—say, English and “Markerese” (e.g., Katz and Fodor, 1963)—he does so because of a strong prior commitment that semantics *as such* implies a relationship between language and the world. Referring to the structured language-like outputs of the ‘projection rules' in a generative semantic model like Katz and Fodor's as “semantic markers,” Lewis writes (p. 18),

Semantic markers are symbols: items in the vocabulary of an artificial language we may call Semantic Markerese. Semantic interpretation by means of them amounts merely to a translation algorithm from the object language to the auxiliary language Markerese. But we can know the Markerese translation of an English sentence without knowing the first thing about the meaning of the English sentence: namely, the conditions under which it would be true. Semantics with no treatment of truth conditions is not semantics.

However, he does point to a way in which he may be okay with such theories; so long as they make a provision for *real* semantics, continuing,

Translation into Markerese is at best a substitute for real semantics, relying either on our tacit competence … as speakers of Markerese or on our ability to do real semantics at least for … Markerese.

Remarkably, Chomsky (1989) appears to think this is precisely how it goes—that the phenomena a Lewisian semanticist is characterizing is a step removed from language proper, writing (p. 324),

[the first] step in the process of interpretation …should be considered to be in effect an extension of syntax, the construction of another level of mental representation beyond LF [‘Logical Form'], a level at which arguments at LF are paired with entities of mental representation, this further level then entering into ‘real semantic interpretation.'

Allowing for such a “two-step” interpretation would allow the theorist to be an internalist about linguistic meaning but an externalist about semantics, if that term is reserved for theories of how expressions (in whatever language) relate to the world.

Pelletier (2011) expresses concern that shifting the work of semantic theory wholly ‘inside the head' would take us too far away from the data on which the theory is based, namely, communication: “For one thing, it is difficult to see how mutual understanding can ever be guaranteed or even achieved with such a view” (p. 33). Worse, “it is hard to see how any truth-conditional account could be involved in conjunction with internalism” about meaning (Pelletier, 2011). Yet, while at least Jackendoff (1984, 1994, 2002) has attempted to show how we might model the first step, only recently have there been stirrings from within the truth-conditional camp that would support two-step interpretation at all. As examples, though, Glanzberg (2014) inches in this direction, supposing that the primary data result from “features of meaning represented within the language faculty, and features of extra-linguistic concepts.” Pietroski (2010) takes things quite a bit further, as noted in some more detail below.

How could this help? If we take the intuitions of semantic anomaly like those in section 1 to indicate something about the relationship between morphosyntactic objects and elements of non-linguistic conceptualization, then it becomes an empirical matter what “scales” amount to—this cannot be stipulated in advance, and it need not track our folk intuitions about what such scales amount to “in the world”^22^. Our metaphysical judgments, just like our metalinguistic judgments, are the subject matters of different disciplines, interlocked in the explanation of how language is understood. More concretely, it will support a view in which the asymmetry between *completely full* and ?*completely tall* is explainable in terms of the nature and structure of the relation between language and conception, while our introspective intuitions about the nature of the associated scales are not.

I will next provide a sketch of how this might look from the perspective of formal semantics. Of primary importance, though, is that a view in which semantics primarily traffics in describing a language-mind connection invites cognitive psychology as a bound “on its right^23^.” With it, we have an independent empirical theory that can restrict the nature and variety of the claims that semanticists can make with respect to what there is^24^.

## 4. Positive Proposal

The approach I urge grounds at least some of our judgments of semantic anomaly in the relation between linguistic and non-linguistic cognition, but it grounds our judgments of truth and falsity in the relation between non-linguistic cognition and the world.

**Figure 1:**
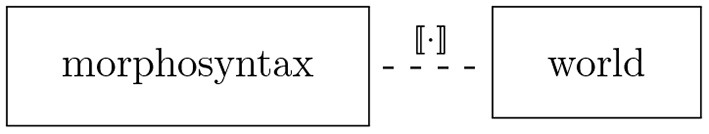
The traditional model in truth-conditional semantics.

**Figure 2:**
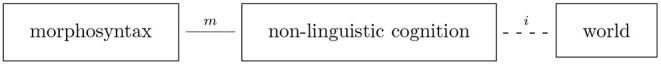
A finer-grained model.

Where the traditional model in truth-conditional semantics (section 3.1) supports a boxology like that in Figure 1, I propose the finer-grained view in Figure 2. Assuming, not without controversy (see footnote 17), that the lines indicate functional relationships between one domain and another, the suggested picture characterizes semantic theories couched in ⟦·⟧ terms as the composition of two functions, here *m* and *i* to evoke “meaning” and “interpretation,” respectively. If all goes well, *i* will do what a truth-conditional semanticist wants ⟦·⟧ to do, but it will assign truth conditions to Thoughts—structured representations internal to the mind that an animal may have quite independently of natural language (cf. Pietroski, 2010)^25^. In contrast, *m* will reveal, at least, the logical properties of natural language expressions and the classes of concepts relevant for their interpretation by *i*. I intend to locate anomalies like those discussed in section 1 at *m*.

Determining whether any given meaning-related phenomenon belongs in one or the other category is not easy. However, it is possible that deeper probing of the nature of different judgments of (un)acceptability and (in)felicity could help. To begin thinking about this, we may first consider the well-known examples in (21) (Chomsky 1957).


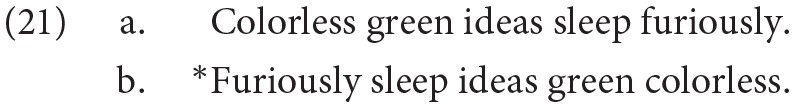


Importantly, (21a) is a well-formed and acceptable sentence of English despite the impossibility of the state of affairs it describes, and the judgment that it is contradictory is almost beside the point. (21b), in contrast, is an ill-formed and unacceptable string of words in English—a thing for which the question of truth or falsity does not arise. Contrasting our target cases, (22a) like (21a) gives us no felt sense of anomaly, yet in my judgment (22a) presents as clearly and distinctly contradictory.


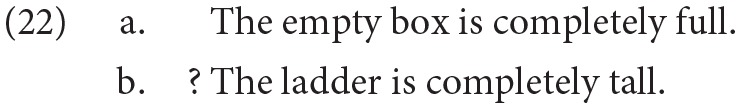


Unlike any of (21a), (21b), or (22a), (22b) is clearly unnatural and unacceptable, but this is apparently not due to any syntactic defect. Importantly, though, the question of truth or falsity does not arise for (22b) just as it does not for (21b).

What is needed, I submit, is a way of thinking about issues with the instructions for concept composition at play in (22b) but not in (21a). Within the general framework I advocate, at least two things must go right at *m* prior to evaluation of truth and falsity at *i*: (i) the sentence must be well-formed according to (at least) the morphosyntactic rules of the language, and (ii) the associated non-linguistic representations or concepts must themselves be well-formed^26^. (21a) and (22a) will, or so I shall suppose, meet both (i) and (ii)^27^. (21a) will run afoul of (i), and, I suggest, (22b) runs afoul of (ii). Such an explanation will require not only the familiar attention to (i) but serious acknowledgment of where the answers to (ii) may be found. How might we get there?

First, we may for simplicity's sake suppose that part of the meaning of lexical items is a “pointer” from within the language system to outside of it (Glanzberg, 2014). Then we can say that what determines whether a lexical item associates with a bounded scale (whether upper or lower) depends on what that lexical item points to and what relations and operations are defined for such concepts. (A central tenet of “core knowledge” approaches in psychology supposes that such knowledge comes in largely domain-specific packages, both in terms of representations and rules; see below.) If *tall* and *wide*, for example, point to a class for which length measures are defined, while *full* points to a class for which such measures are not defined, the explanation for the asymmetry in (4), repeated as in (23), can be explained in terms of these independent posits: (23b) invites the construction of a complex concept that cannot be evaluated for truth or falsity.


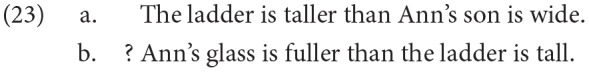


Second, we must take quite seriously the types of restrictions that semanticists like lay down for the compositional requirements of expressions like *completely*, but understand them in a different way than previously. I suggest that we understand these requirements in terms of restrictions on the composition of concepts. More concretely, Kennedy and McNally (2005) suppose that (24) is a reasonable approximation of the semantic contribution of this modifier.


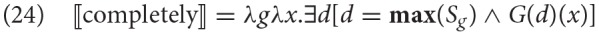


As those authors write, “Assuming that the **max** function returns a value only for scales with maximal values, this modifier can combine only with gradable adjectives that have scales that are closed on the upper end” (p. 369). In the present framework, we may understand these specifications as restricting the space of concepts that *completely* can compose with. For a complete theory, we will want to know, of course, how to distinguish the concepts that are so composable from those that are not—and for this, we must turn to cognitive psychology.

To test our theories, we must take a hard and independent look at the neighboring cognition, as this is where empirical evidence for the nature and compositional structure of concepts can be sought. An easy place to start, I submit, is the cognitive and developmental psychology literature on core knowledge (for example, Spelke, 1998, 2003; Carey, 2009, and many others; see Strickland, 2016 for a related view)^28^. We know from this literature that, from the earliest stages of the development of humans as well as that of many other species, there exist domain-specific faculties of mind that undergird our intuitive understanding of what there is and how things work across a host of contentful categories: objects, events, time, causation, agency, and more. The knowledge that partly constitutes each of these faculties is both highly specific and uniform across the species^29^, and it is reasonable to suppose that the initial conceptual repertoire it provides restricts the available concept composition operations and scaffolds all later concept acquisition.

If we understand the formal statements of our semantic theory as encoding, in part, hypotheses about the kinds of representations and structures available in extralinguistic cognition, then we can test its predictions against what we know independently about extralinguistic cognition. In some cases, this can mean leveraging formal semantics as a source of hypotheses about representation. If the thematic or participant structure of events is important for a semantic theory, we can probe the nature and structure of our nonlinguistic event concepts in nonlinguistic tasks (e.g., Wellwood et al., 2015). If our theories require a privileged difference between object and substance predications, we can leverage the cognitive object/substance distinction (e.g., in the evaluation of *more* NP, see Barner and Snedeker, 2004; Odic et al., 2018). Where our theories say that the formal structure of objects and events is parallel, we can find ways of evaluating the psychological plausibility of the parallelism independently of language (e.g., Wellwood et al., 2018a).

One such arena of particular relevance for degree semantics is the literature on magnitude estimation, in which the Approximate Number System (ANS) is the most lauded^30^. The ANS is an evolutionarily ancient system that generates percepts of “numerosity,” demonstrably in place in human children within the earliest time window in which it is possible to test (see especially Dehaene, 1997; Feigenson et al., 2004). Now, while ANS representations are ordered Gaussian distributions, which look different on the face from the set of discrete, ordered points required for cardinality comparisons in natural language, these two “scales” are isomorphic (e.g., Gallistel and Gelman, 1992; cf. Odic et al., 2015). And indeed, there is ample evidence that while the careful evaluation of a sentence like *Most of the dots are blue* tracks precise cardinality, speeded evaluation shows signs of the ANS (within and across individuals, across development, and across languages; see e.g., Halberda et al., 2008; Hackl, 2009; Pietroski et al., 2009; Lidz et al., 2011; Tomaszewicz, 2011).

In this light, we may consider how to address crosslinguistic differences like those noted in section 3, e.g., the apparent coextensivity of *spaghetti* and *noodles* despite their hypothetically distinct commitments to stuff vs. plurality. When English speakers use *spaghetti* as a mass term and Italians use it as a plural term, are they really thinking about what is on the plate differently?^31^ This is an empirical question that can be tested. For example, if plural predications must be evaluated by number with *more* (see Wellwood, 2018 and references therein) while mass predications can but need not (see Barner and Snedeker, 2005 for experimental evidence), then we might expect *more spaghetti* to show more flexibility in its evaluation in English than in Italian when (say) number and volume are available as orthogonal options. Yet, we might appreciate a common *perception* by investigating how speakers view the images *independently of language* by constructing a comparable task that renders linguistic encoding unusable, e.g., by comparing similarity judgments of the same pairs of images, delivered while performing verbal shadowing^32^.

On this general approach, the mismatches between our semantic and metaphysical intuitions pointed out in section 1 can be accommodated; since we distinguish the relations *i* and *m*, we may find restrictions in place at *m* (tracking our semantic intuitions) that are determined independently, and perhaps antecedently, to whatever we know at *i* (tracking our metaphysical intuitions). Recall, for example, the issue that our intuitive sense of the scale of cost—hypothetically that which is associated with adjectives like *expensive*—has a minimum element but *slightly expensive* does not mean what it should if the modification theory is correct. Our intuitions about what would count as a minimal element track *i*, but the anomaly we detect occurs already at *m*.

Importantly, a model of interpretation in two steps supports an account of polysemy^33^, in which a single pointer (at *m*) involves a choice of resolution for the concept ultimately “fetched” (hence determining the input to *i*; e.g., Pietroski, 2018). Pelletier's (2011) worry about “the” semantic value of *water* could thus evaporate: we may have some early, core concept that we associate with the word but a different one after we do some science. Our early conceptual repertoire plays an important role in our cognitive economy throughout our lives and is likely responsible for endowing us with a naive concept of water that meets the divisiveness condition (cf. Prasada et al., 2002; Wellwood et al., 2018b). However, this repertoire does not restrict us from acquiring new concepts—e.g., one that is identical in extension with that of *H*_2_0—even if the two may ultimately be in conflict, metaphysically. This added degree of flexibility can similarly provide an angle on some of the cases discussed in the first half of the paper: perhaps language is wired by default to the sorts of concepts given to us biology—itself a matter of empirical discovery—that can differ from those that we acquire through reflection or experience.

Thus, our semantic intuitions might track properties of conception that are below what is available to introspection, while our metaphysical intuitions reflect a composite of (and sometimes tension between) our naive concepts and our more reflective ones.

## 5. Conclusion

I considered a case study in degree semantics and scale structure, leveraging putative counterexamples in this arena to advocate for a finer-grained model of semantic interpretation than is traditionally supposed within truth-conditional frameworks. Specifically, I offered the view that we can make sense of these counterexamples by assuming a model that divides interpretation into (at least) two steps. Semanticists are not in the business of formulating statements about how expressions compositionally relate to entities in the world but about how they compositionally relate to representations and operations in non-linguistic cognition. The outputs of the first step of interpretation—*m*—may themselves be submitted to truth-conditional evaluations that depend on what the world is like.

Semantic theory cannot only attend to what we talk as if there is, on pain of being rendered non-scientific. Instead, the two-step program integrates semantics within a tapestry consisting of necessary interdisciplinary links, wherein not only morphosyntactic theory but theories of conceptual structure inform theories of meaning and vice versa. As a bonus, the two-step program offers the kind of latitude that can support matters of polysemy, which will minimally be required for a complete account of the meaning of modifiers like *completely* (in addition to their guise as maximizers, they function as markers of confidence, etc.) More importantly, the possibility of a given lexical item pairing with more than one concept can help explain mismatches between our semantic and metaphysical intuitions.

The resulting view positions semantic theory as having a crucial role in furthering our understanding of the ways that the mind structures its experience. Semanticists theorize about all kinds of things that expressions may be “about”—in addition to objects, substances, and times, we posit events, processes, states, negative events, possible worlds, impossible worlds, and so on. Much of this talk would be news to psychologists, though there are already good case studies illustrating the payoffs for cognitive psychology of testing semantic posits as hypotheses about representation (for a very recent example, see Wellwood et al., 2018b). The approach I advocate thus invites semanticists to explicitly characterize their theory in such a way that it may be tested by these neighboring fields, and it invites psychologists to read our theories this way even when not so-intended. In this way, semantic theory can finally vindicate the idea that language is “a window into the mind.”

## Author Contributions

The author confirms being the sole contributor of this work and has approved it for publication.

### Conflict of Interest

The author declares that the research was conducted in the absence of any commercial or financial relationships that could be construed as a potential conflict of interest.

## Footnotes

^1^Degree-based theories have dominated recent discussion of structures like (1) and (2) as well as those of comparative sentences with *more, as*, etc. Degree-based theories contrast with delineation-based theories (see Burnett, 2016 for discussion and citations), but I only address the former here.

^2^In English at least; (see, among others, Beck et al., 2004, 2010; Bogal-Allbritten, 2013; Bochnak, 2015).

^3^In this paper, the diacritics on sentences reflect my choices which, in some cases, differ from those of the authors whose work is under discussion. “?” is used throughout to indicate a felt anomaly related to meaning; when any other diacritics are used, flags are supplied to indicate how are they are meant to be interpreted.

^4^The difference between *tall* and *full* is often referred to as the relative/absolute distinction in gradable adjectives, and was apparently first noticed by Unger (1971). Not all of the relevant tests are reviewed here; see Rotstein and Winter (2004), Kennedy and McNally (2005), Lassiter (2011, 2017), and Klecha (2014) for tests involving proportional modification with *half, 90%*, and *mostly*, entailments between sentences with positive adjectives and their intuitive antonyms, and others. Of note in light of the squishiness of judgments in this domain is Kennedy's (2007) suggestion that *perfectly* (maximizer) and *slightly* (minimizer) are generally best at showing the relevant interpretive patterns across a broad array of gradable adjectives (Kennedy, 2007, p. 34).

^5^Of course, we assume that speakers are generally competent in their language and know what the expressions in their language are about (or, at least, linguists tend to assume this); compare, for example, Putnam (1975) and Chomsky (1995).

^6^Sassoon (2011) hypothesizes that *slightly* works differently—roughly, it picks out different standards depending on whether the property denoted by the adjective is stable (i.e., individual-level) or not (i.e., stage-level)—by noting that all relative adjectives seem to have an intuitive zero, including that associated with *(in)expensive*. Extended consideration of Sassoon's interpretation for *slightly* would complicate the narrow point I want to make in the text but could itself be used to raise the same general issue: how should we independently determine what counts as a stable vs. non-stable property, independently of linguistic tests like compatibility with adverbs like *frequently, rarely*, etc.? See also Toledo and Sassoon (2011) and Solt (2012).

^7^Lassiter, for his part, supposes not that the tests fail to show what they purport to show but just that the relevant generalizations are weaker than their architects supposed: the inference from *completely A*, for adjective *A*, to a scalar endpoint holds, but *A*'s associating with a scalar endpoint does not guarantee the felicity of *completely A*. In other words, the generalization is a conditional one rather than a biconditional one. (Lassiter and Goodman, 2013 offer a very different approach to the relative/absolute distinction.) If so, this would not be so surprising, though it does pose its own explanatory challenges. The situation is analogous to that in the mass/count literature with respect to lexical specifications being overridden by, for instance, the semantic commitments of plural morphology (see e.g., Gillon, 1992, 2012).

^8^Lassiter notes some of the many senses that the maximizing modifier *completely* can take on, obscuring the results of those tests: it can function as a marker of “emphasis, correction, or high speaker confidence” (Lassiter, 2011, p. 13). Beltrama's (2018) study provides an interesting contrast between *completely* and *totally*, which both have maximizing uses, but the latter expresses subjective intensification with adjectives that *completely* sounds awful with; compare ?*completely* tall and *totally tall*.

^9^For more detailed overviews of degree semantics, see Kennedy (2006), Schwarzschild (2008), Wellwood (2019), chapter 2.

^10^“Regular” comparatives like *Ann is taller than Bill is* represent the identity case, where *A* = *A*′= *tall* (see Bresnan, 1973).

^11^In Kennedy's technical implementation, given some privileged point *d*, a positive degree interval is one that starts at the scalar minimum and extends up to *d*, while the (near-) complementary negative degree interval is one that begins at *d* and extends upwards to infinity. This implementation is at odds with some of the details of later developments in modeling scale structure; see below.

^12^This is a species of triviality argument: since, in virtue of its syntax-semantics correspondences, such a sentence will never evaluate to true or false, it is unacceptable; see Gajewski (2002) for extended discussion of this type of theoretical reasoning.

^13^Büring (2007) points out that the phenomenon of “cross-polar anomaly” is actually somewhat more restricted, noting that it only occurs if the negative adjective appears in the *than*-clause; contrast (13) with *The doorway is shorter than the ladder is long*, which is reported to be acceptable. Büring suggests not that the lack of anomaly is a counter-example to Kennedy's theory, but that it reveals syntactic decomposition of negative adjectives; see his paper for details.

^14^Recent research has toyed with revising this basic assumption, analyzing gradable adjectives in terms of properties of states rather than in terms of degree functions (see Fults, 2006; Wellwood, 2012, 2015; Baglini, 2015; Pasternak, 2017; Cariani et al., 2018; Glass, 2019).

^15^I say “bare” because it does not appear with a lexical adjective or adverb. In (15a), there is a “nominal comparative,” but the facts are parallel for verbal comparatives, as I show below. Such cases plausibly involve a functional quantificational element corresponding to (some occurrences of) English *much*, which plays the role of introducing measure functions; cf. Bresnan, 1973; Wellwood et al., 2012.

^16^This interpretation dovetails with some of the few explicit statements of what such semantic theories are committed to: for example, Higginbotham (1985) writes, “Semantic theory proceeds from assumptions both about the nature of syntactic structures and about the nature of semantic values” (p. 553), and Bach (1986) writes, “I understand ‘semantics' in the sense of a theory of the relationship between language and something that is not language” (p. 574).

^17^In this framework, semantic theory describes ⟦·⟧:*E* → *Z*, *E* the set of morphosyntactic objects, *Z* of worldly entities. This is so in the Montagovian tradition á la Heim and Kratzer (1998), where I draw ⟦·⟧ from; a weaker, relational (but still truth-conditional) approach is taken in the Davidsonian tradition á la Larson and Segal (1995), where the relevant relation is called “Val”; see also Martin (1958), as well as works by Higginbotham, Boolos, Pietroski, and Schein (Schein p.c.).

^18^A *Frontiers* reviewer points out that that there may be language-internal reasons why certain (classes of) expressions behave in unexpected ways, for instance constraints on how lexicalization carves up conceptual space (see footnote 23). However, the possibility of recruiting such explanations will plainly depend on one's foundational assumptions, which leads us right back to the present matter of which foundations we should accept for the purposes of semantic theorizing.

^19^This worry applies if we interpret the divisiveness condition very strongly, such that any divisive N, if it applies to some stuff, also applies to any arbitrary subpart of that stuff. See Bunt (1979) and Champollion (2017), among others, for important discussion.

^20^See Partee (2018) for discussion of the history of tension here; she cites Jackendoff (1983) and Fodor (1975) as early exemplars of the current position.

^21^For an accessible introduction to basic concepts in the computational theory of mind and the consequences of a representational format in particular (see Gallistel and King, 2010).

^22^For a recent study of early links between scalar language and independent cognitive systems for representing magnitudes (see Odic, 2018).

^23^Of course, the assumption that language relates to other faculties of mind is explicit in cognitive semantic approaches. Gärdenfors (2014) (and in many of his antecedent works) argues that lexicalization patterns are linked inextricably with the regions or bundles of regions in conceptual space. Assuming a sufficient independent theory of conceptual space, such a theory will make broad predictions about the sorts of meanings we expect to see lexicalized in human languages. Partee has long maintained that inattention to the lexicon in formal semantics is due to the fact that the problems of compositional or structural aspects of meaning are more tractable (see her 1980; 2018, for example).

^24^The following thought occurs to me, though it was not likely offered with an internalist conception in mind: “If we were to think of logic as relating to the structure of thought and of metaphysics as relating to the structure of reality, then logic would provide us with the most general traits of thought and metaphysics with the most general traits of reality” (Fine, 2012, p. 18). The semanticist's data concerning truth/falsity and entailment patterns seem to reveal, indeed, that natural language has a certain logic; I suggest that anomaly like the kinds of cases considered in this paper reveals (at least) our intuitive metaphysics.

^25^Pietroski argues that a number of features of language design can be explained in terms of a contrast between the format of linguistic meanings and extralinguistic Thoughts. A central example is that of predicate adicity: while much research in formal semantics suggests a monadic, eventive interpretation of verbs like *give* (i.e., λ*e*.**give**(*e*)), there is evidence to suggest that the Thought associated with *give* sentences should nonetheless be analyzed with a triadic structure [involving, e.g., GIVE(*x, y, z*)]; see his 2010; 2018. See also Gordon (2003), who provides evidence for the adicity of such event concepts in prelinguistic infants.

^26^Alternatively: the “instructions” for constructing those representations or concepts must be evaluable.

^27^If the difference in salience regarding their contradictoriness is real, one possibility is that this is due to the compositional distance between the pieces that compose to deliver the contradiction. In (21a), this point arrives as soon as *colorless* and *green* come together, for example, whereas in (22a), it arrives only once the subject is composed with the predicate.

^28^Partee (2018) seems to have a similar sort of investigation in mind, writing “…if we follow Burge (2010) in drawing insight from how perception works and how it gives (fallible) veridical knowledge prior to any “reasoning,” we can see semantics, including at least parts of the difficult area of lexical semantics, as a particularly important and fruitful branch of psychology,” suggesting further that “philosophy of language *need[s]* philosophy of mind for a resolution of some apparent problems in the foundations of semantics” (p. 190; her emphasis).

^29^Indeed, they are likely responsible for our apparently species-level construction of a common experiential world (e.g., Jackendoff, 1994; Hoffman, 2009).

^30^Connections between the grammar of comparatives and the cognitive resources of magnitude estimation and comparison was suggested in quite another context in Fox and Hackl (2006).

^31^Incidentally, noteworthy Italian speaker Paolo Santorio, p.c., answers this question with a resounding “yes!”.

^32^See Wellwood et al. (2016) and Wellwood et al. (2018b) for the use of a similarity-judgment task to get at the salience of numerical differences and Spelke (2003) for important results gleaned from verbal shadowing tasks.

^33^See, in this connection, Pietroski (2010) and Vicente (2012) for recent discussion, novel approaches, and citations to relevant literature.
